# Dataset of differentially regulated proteins in HUVECs challenged with wild type and *UGM1* mutant *Aspergillus fumigatus* strains

**DOI:** 10.1016/j.dib.2016.07.062

**Published:** 2016-08-18

**Authors:** Gabriela Westerlund Peixoto Neves, Nathália Curty, Paula Helena Kubitschek-Barreira, Thierry Fontaine, Gustavo Henrique Martins Ferreira Souza, Marcel Lyra Cunha, Gustavo H. Goldman, Anne Beauvais, Jean-Paul Latgé, Leila M. Lopes-Bezerra

**Affiliations:** a Universidade do Estado do Rio de Janeiro, Campus Maracanã, Pavilhão Haroldo Lisboa da Cunha sl 501D, CEP: 20550-013, Rio de Janeiro, RJ, Brazil; b MS Applications Research and Development Laboratory, Waters Corporation, São Paulo, Brazil; c Unité des Aspergillus, Institut Pasteur, 25 rue du Docteur Roux, 75724, Paris Cedex 15, France; d Universidade de São Paulo, Faculdade de Ciências Farmacêuticas de Ribeirão Preto, Departamento de Ciências Farmacêuticas. Av. do Cafe S/N, Monte Alegre, CEP:14040-903, Ribeirao Preto, SP, Brazil

**Keywords:** *Aspergillus fumigatus*, HUVEC, GAG, *UGM1* mutant, PROTEOME, Invasive aspergillosis

## Abstract

Invasive aspergillosis is the primary opportunistic invasive fungal infection described in neutropenic hematologic patients, caused by the angioinvasive pathogen *Aspergillus fumigatus*. The molecular mechanisms associated with *A. fumigatus* infection in the vascular endothelium are poorly understood. In this context, we used a high-throughput proteomic approach to unveil the proteins modulated in HUVECs after interaction with a wild type strain and the *UGM1* mutant (Δ*ugm1*) of *A. fumigatus*. The proteomic analysis was also performed in HUVECs challenged with a galactosaminogalactan (GAG) purified from *A. fumigatus* cell wall. The dataset presented here correspond to all proteins identified that fit a 2-fold change criteria (log 2 ratio ≥ 1 or ≤ −1), disregarding the statistical validation cut off, in order to supplement the research article entitled “Modifications to the composition of the hyphal outer layer of *Aspergillus fumigatus* modulates the HUVEC proteins associated with inflammatory and stress responses” (G.W.P. Neves, N.A. Curty, P.H. Kubitschek-Barreira, T. Fontaine, G.H.M.F. Souza, M. Lyra Cunha, G.H. Goldman, A. Beauvais, J.P. Latgé, L.M. Lopes-Bezerra, 2016) [1]. The mass spectrometry proteomic data have been deposited in the ProteomeXchange Consortium via the PRIDE partner repository with the dataset identifier PRIDE: PXD002823.

**Specifications Table**

**Table:** 

Subject area	*Biology*
More specific subject area	*Medical Mycology*
Type of data	*Table, image, figure*
How data was acquired	*Scanning electron microscope JEOL JSM-6510 LV, Fluorescence Microscope, nanoACQUITY UHPL System Waters, Synapt G2-S Waters.*
Data format	*Filtered, Analyzed*
Experimental factors	*Confluent HUVEC monolayers were infected with thimerosal-treated germlings of the wild type and Δugm1 strains of Aspergillus fumigatus and the purified GAG. After 16 hours of infection, an extract of whole HUVEC protein was obtained.*
Experimental features	*All HUVEC protein extracts were analyzed using a 2D-nanoLC-HDMS*^*E*^*approach.*
Data source location	*Rio de Janeiro, Brazil*
Data accessibility	*Data is within this article and deposited in the ProteomeXchange Consortium via the* PRIDE partner repository with the dataset identifier PRIDE: PXD002823.

**Value of the data**

• This dataset will be of value to the scientific community aiming to analyze the identified proteins modulated in HUVECs upon infection by *A. fumigatus* and the participation of the cell wall galactosaminogalactan (GAG) in endothelial activation.
• This data article contributes to the scientific research concerning the role of GAG in the physiopathology of invasive aspergillosis.
• The data provided here identify key proteins involved in the response of HUVECs to different strains of *A. fumigatus*, contributing to the knowledge of angioinvasive molecular mechanism in invasive aspergillosis.

## 1 Data

**Fig. 1:**
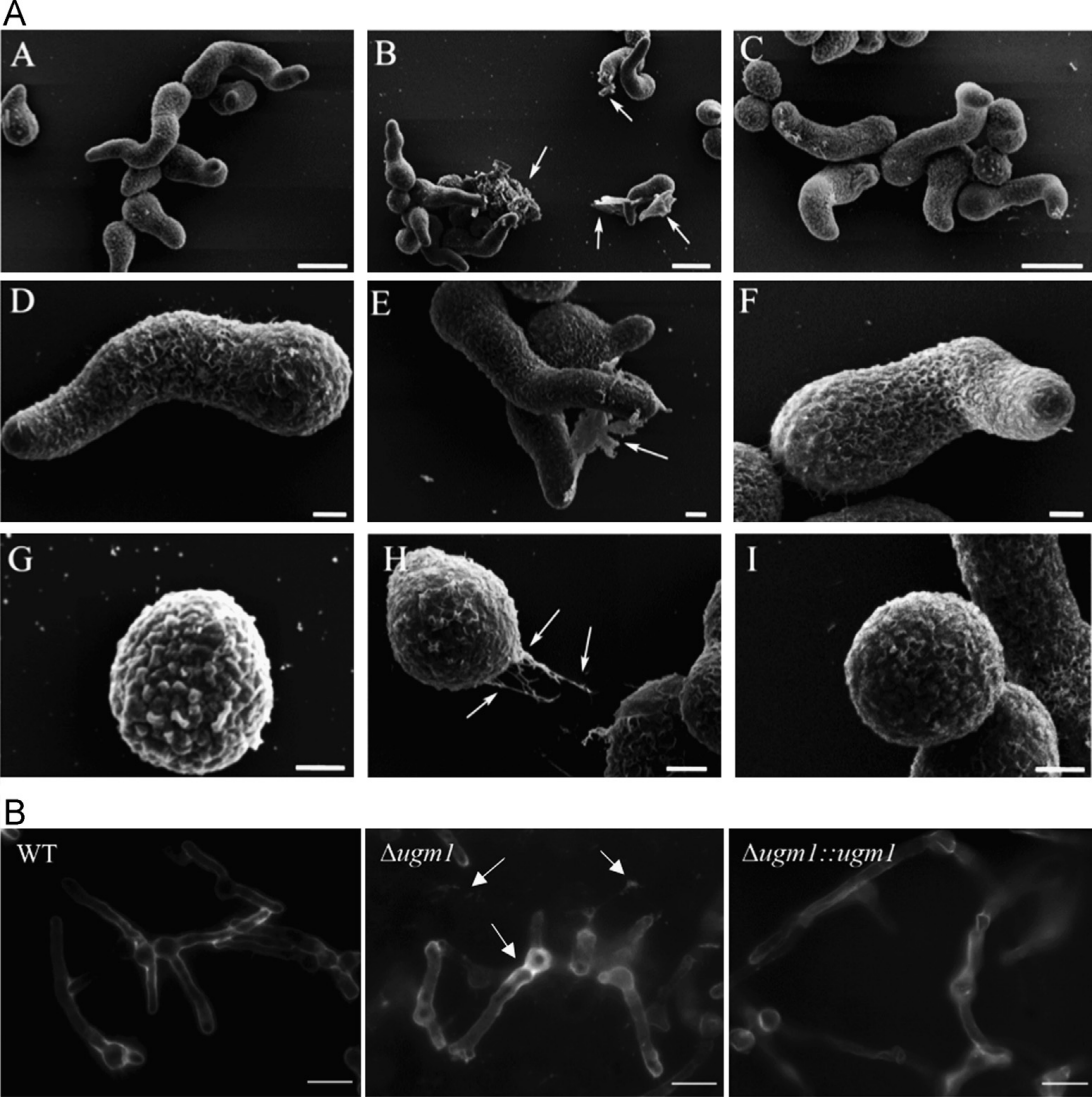
**A** The germlings (A–F) and swollen conidia (G–I) of *A. fumigatus* wild type (A, D and G) Δ*ugm1* (B, D and H) and Δ*ugm1:ugm1* (C, F and I). Δ*ugm1* showed increased production on an amorphous thin substance typical of *A. fumigatus* ECM (arrows). Swollen Δ*ugm1* conidia (H) released a filament from the wall that apparently adhered to the coverslip (arrows). Bars: A–C 5 µm. D–I =1 µm. **B**: Detection of galactosaminogalactan using immunofluorescence on germinated conidia. The mutant showed an increased amount of GAG on the cell wall of germinated conidia and an adherent secreted material in the Δ*ugm1* (arrows). Bars: 10 µm.

**Fig. 2:**
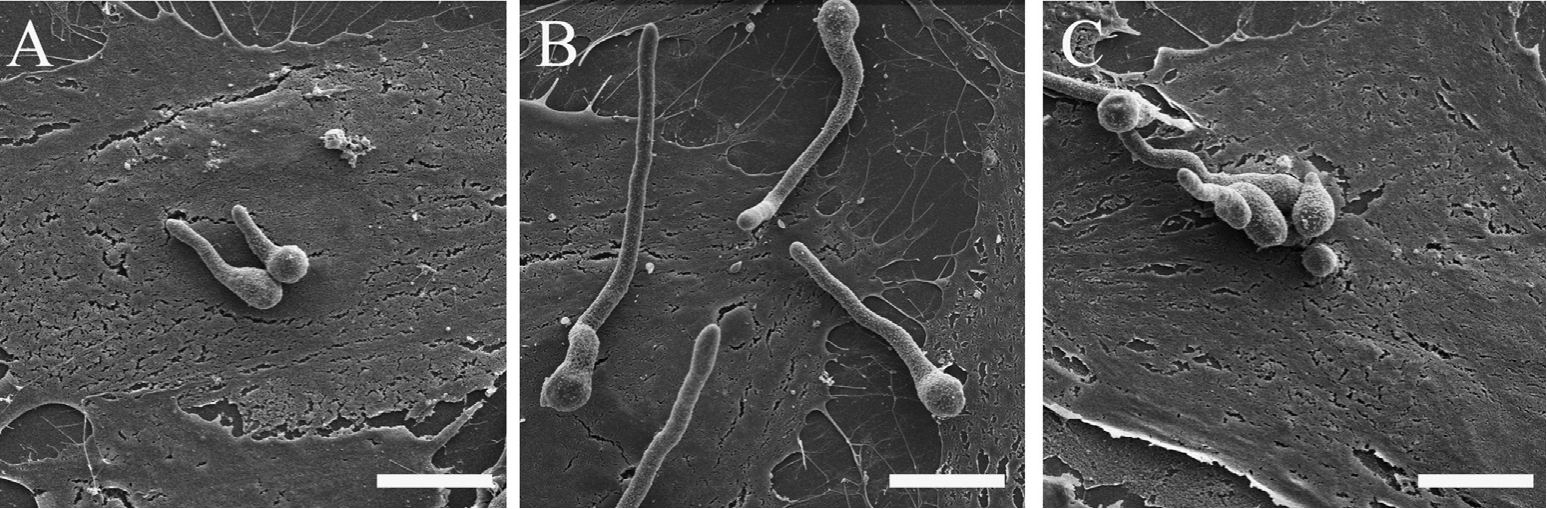
SEM of HUVECs monolayers challenged with the germlings of different strains of *A. fumigatus.* HUVEC interaction with the wild type (A), Δ*ugm1* (B) and Δ*ugm1:ugm1* (C) strains. Bars: A–C 5 µm.

**Fig. 3:**
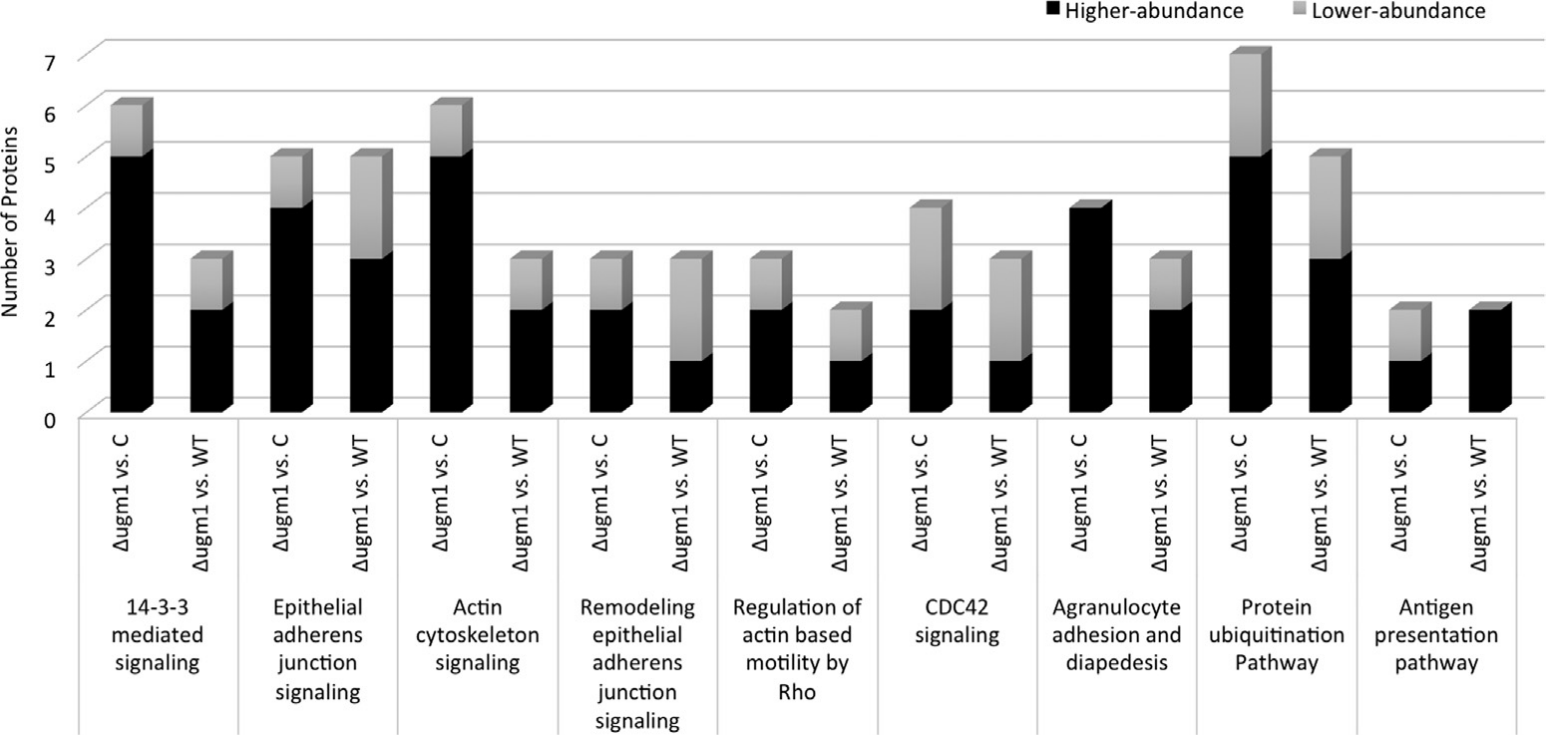
Ingenuity pathway analysis (IPA) showing the commonly regulated HUVEC routes of the ∆*ugm1* mutant of *A. fumigatus* based on the following comparisons: ∆*ugm1* vs. uninfected control and ∆*ugm1* vs. WT. The graph shows the main routes regulated through Δ*ugm1*, indicating the number of proteins with higher-abundance (black) and lower-abundance (gray) levels. For this analysis, we used the quantitation criteria of a minimum 2.0-fold change.

**Fig. 4:**
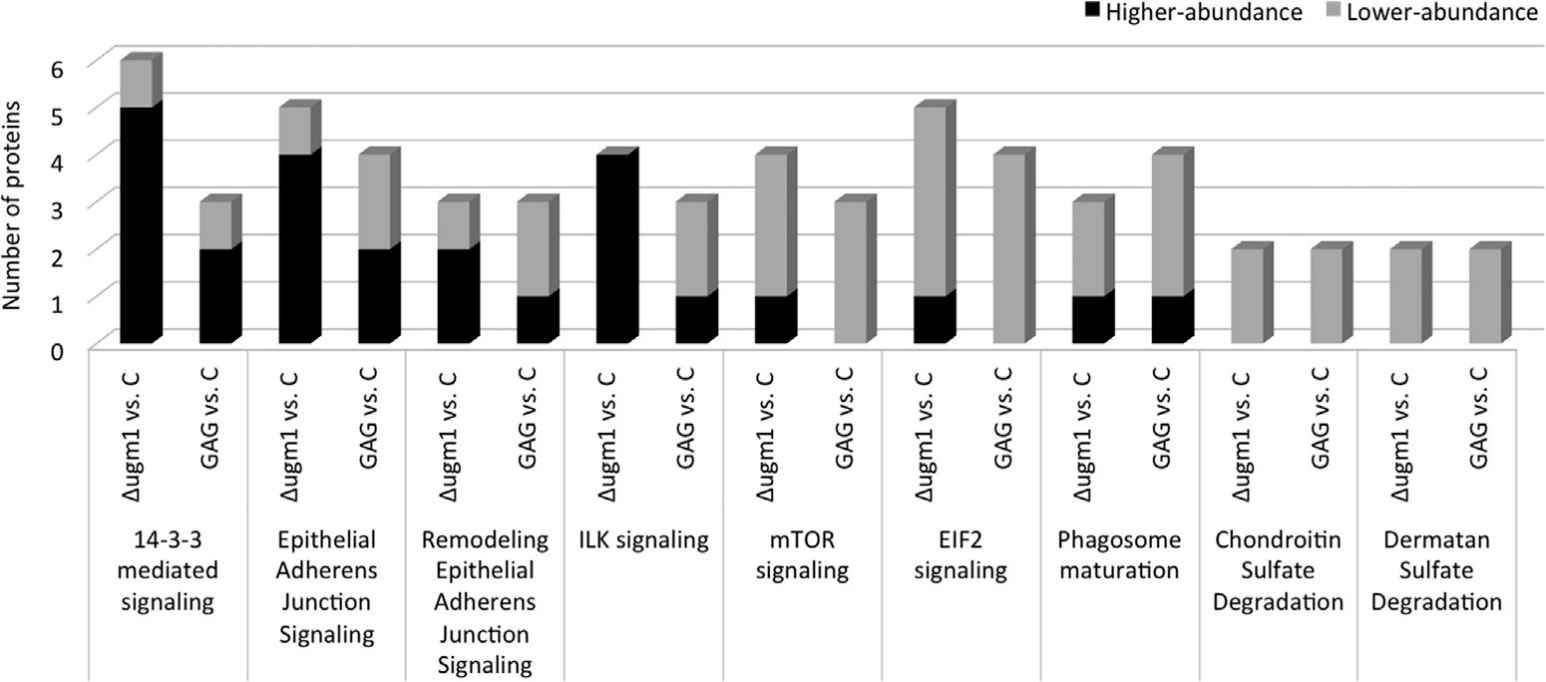
Ingenuity pathway analysis (IPA) showing the HUVEC routes commonly regulated through GAG and the ∆*ugm1* mutant compared with the HUVEC uninfected control. The graphic shows the number of proteins with higher-abundance (black) and lower-abundance (gray) levels. For this analysis, we used the quantitation criteria of a minimum 2.0-fold change.

**Fig. 5:**
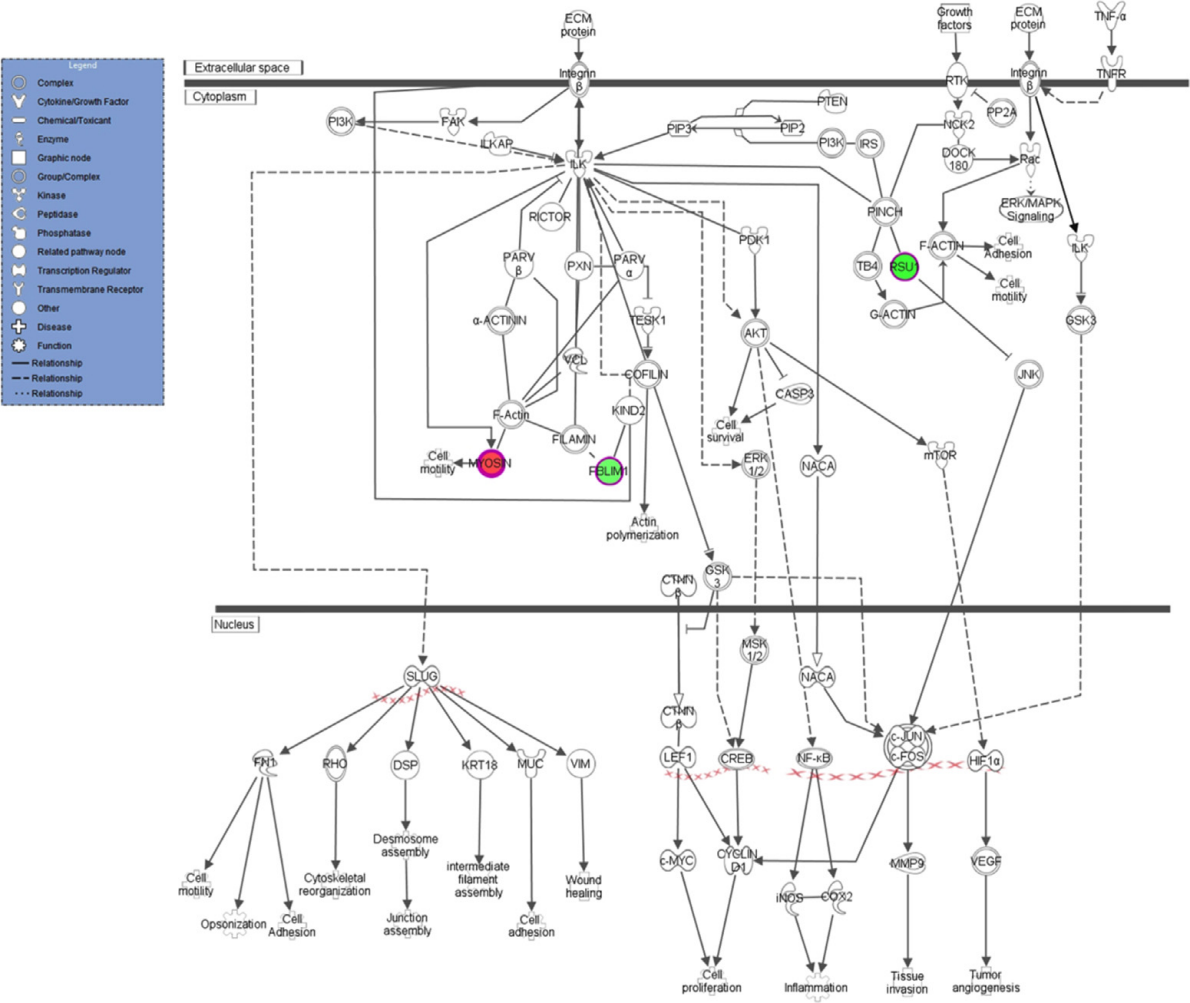
Illustration of the ILK signaling pathway generated using QIAGEN׳s Ingenuity^®^ Pathway Analysis, differentially regulated under GAG interaction conditions. The proteins in red and green indicate higher and lower abundance levels under GAG interaction conditions, respectively. In addition, the figure also illustrates that TNF-α participates in the upstream regulation of this pathway. The arrows indicate predicted regulation, activation (→) or inhibition (⊢). The proteins used in this analysis were filtered using the following criteria: minimum of 2.0-fold change and quantitation ANOVA, *p*-value ≤ 0.05. ECM protein-extracellular protein; FAK - Focal adhesion kinase; PI3K − 1 Phosphatidyl inositol 3 kinase; ILKAP - Integrin-linked kinase-associated serine/threonine phosphatase 2C; ILK - Integrin-linked protein kinase; PARVβ- Parvin beta; RICTOR - Rapamycin-insensitive companion of mTOR; PXN – Paxilin; VCL – Vinculin; Filamin-binding LIM protein 1; KIND2 - Fermitin family homolog 2; TESK1 - Dual specificity testis-specific protein kinase 2; PARVα - Parvin alpha; PDK1 − 3-phosphoinositide-dependent protein kinase 1; AKT - Protein Kinase B; CASP3 - Caspase-3; PIP3 - Phosphatidylinositol (3,4,5)-trisphosphate; PIP2 - phosphatidylinositol biphosphate; PI3K − 1 Phosphatidyl inositol 3 kinase; PTEN - Phosphatidylinositol 3,4,5-trisphosphate 3-phosphatase and dual-specificity protein phosphatase PTEN; IRS - Insulin receptor substrate; PINCH - LIM and senescent cell antigen-like-containing domain protein; TB4 - Thymosin beta-4; RSU1 - Ras suppressor protein 1; RTK - Receptor tyrosine kinase; PP2A - Protein phosphatase 2A; NCK2 - Cytoplasmic protein NCK2; DOCK 180 - Dedicator of cytokinesis protein 180; TNFR - tumor necrosis factor receptor; GSK3 - Glycogen synthase kinase-3; JNK - c-Jun N-terminal Protein Kinase; mTOR - Serine/threonine-protein kinase mTOR; NACA - Nascent polypeptide-associated complex subunit alpha; ERK 1/2 - Mitogen-activated protein kinase ERK 1/2; CTNNβ - Catenin (Cadherin-Associated Protein), Beta; MSK 1/2 - Mitogen and stress activated protein kinase 1/2; CREB - Cyclic AMP-responsive element-binding protein; LEF 1- Lymphoid enhancer-binding factor 1; cJUN – Jun Proto-Oncogene; cFOS – FBJ Murine Osteosarcoma viral oncogene homolog; HIF 1α - Hypoxia-inducible factor 1-alpha; c-MYC - Myc proto-oncogene protein; iNOS - Nitric oxide synthase, inducible; COX2 - Prostaglandin G/H synthase 2; MMP9 - Matrix metalloproteinase-9; VEGF - Vascular endothelial growth factor; SLUG - Zinc finger protein SNAI2; FN1 – Fibronectin; RHO - Ras-like GTP-binding protein Rho; DSP – Desmoplakin; KRT 18 - Keratin, type I cytoskeletal 18; MUC – Mucin; VIM – Vimentin. (For interpretation of the references to color in this figure legend, the reader is referred to the web version of this article).

Fig. 1 shows an increased GAG cell wall expression by the ∆*ugm1* strain of *A. fumigatus* and, Fig. 2 further illustrates the interaction of HUVECs monolayers with germlings of all *A. fumigatus* strains. Supplementary tables 1–3 present the respective list of identified HUVEC proteins, including the difference in the abundance levels, in the following interaction conditions: ∆*ugm1* vs. control; WT vs. control; ∆*ugm1* vs. WT; and GAG vs control. Fig. 3, Fig. 4, Fig. 5 show the pathways that were predicted by Ingenuity Pathway Analysis (IPA) modulated in HUVECs challenged with the *UGM1* mutant and the purified cell wall GAG.

## 2 Experimental design, materials and methods

### 2.1 Strains, culture conditions and microscopic analysis of the fungal cell surface

The Ku80 pyrG^+^ parental strain (WT) [2], the *UGM1* mutant (*Δugm1*) and the reconstituted strain (*Δugm1:ugm1*) [3] were cultivated as previously described [1], [4]. The purified urea-soluble fraction of galactosaminogalactan (GAG) was obtained as described [5].

For scanning electron microscopy (SEM), the conidia were placed on glass coverslips coated with 2% gelatin and incubated in Sabouraud broth for 6 h at 37 °C, to obtain the germ tubes (germlings). After, germlings were washed in PBS and fixed for 1 h with 2.5% glutaraldehyde plus 4% paraformaldehyde in 0.1 M calcodylate buffer (pH 7.2), washed three times with the same buffer and post-fixed with 1% osmium tetroxide. The cells were subsequently dehydrated in a graded ethanol series and critical point-dried with CO_2_. The specimens were coated with gold in a Balzers sputtering apparatus and the micrographs were captured using a JEOL JSM-6510 LV SEM. The images were processed using ImageJ software. The obtained scanning electron microscopy (SEM) images of germlings of different *A. fumigatus* strains are shown in Fig. 1A. This experiment was performed twice with experimental duplicates.

For GAG immunolabeling, the conidia were germinated overnight in Brian׳s medium [6] on eight-well Permanox chamber slides (Lab-Tek^®^) at 30 °C and further fixed with 2.5% (w/v) paraformaldehyde for 2 h at room temperature. After fixation, the cells were washed with 0.1 M NH_4_Cl in PBS for 5 min, followed by incubation with 5% goat serum in PBS for 1 h. The cells were incubated with an anti-galactosaminogalactan monoclonal antibody (kindly provided from Dr. Jean-Paul Latgé, Pasteur Institute, Paris) at 20 mg/ml in 5% goat serum/PBS for 1 h at room temperature. After washing with goat serum/PBS, the cells were incubated with goat TRITC-conjugated anti-mouse IgG(H+L) (Sigma) diluted 1:200 in goat serum/PBS. After washing with PBS, hyphae of *A. fumigatus* strains were visualized with a fluorescence light microscope, and the images are shown in Fig. 1B. A mock monoclonal antibody was used as a control. This experiment was performed three times with experimental duplicates.

### 2.2 Culture of human vein endothelial cells (HUVEC) and interaction assay

HUVECs were obtained as previously described in [1], [7]. The use of HUVECs was approved through the Research Ethics Committee of the Municipal Health Secretary and Civil Defense of Rio de Janeiro (CEP SMSDC-RJ), protocol no. 196/09. In total, 18 volunteers participated in the present study.

For the interaction assays, HUVEC monolayers were infected with germlings of *A. fumigatus* WT and ∆*ugm1* strains, and with purified GAG (1 µg/mL), as described previously [1]. Uninfected HUVEC monolayers (control) were maintained in the same culture conditions. For SEM the HUVEC assays were performed as described above, section 1.1. The SEM images of HUVEC monolayers challenged with germlings of all *A. fumigatus* strains are shown in Fig. 2.

### 2.3 Label-free protein analysis using mass spectrometry, database searching and quantification

The proteins extracts used for proteomics assays were obtained as follows. The endothelial cell monolayers cultivated on 75-cm^2^ culture flasks were infected with thimerosal-killed germlings of *A. fumigatus* [4] or were interacted with the purified GAG. After, HUVEC monolayers were washed twice with Hank´s balanced salt solution (Cultilab) and gently harvested using a cell scraper. Next, the cells were centrifuged at 200 g, for 10 min. The pellet was subsequently suspended in 250 µL of a lysis buffer (8 M urea, 1 M Tris, 4% (w/v) CHAPS, supplemented with 1 mM PMSF, 5 mM EDTA, 160 µM leupeptin, 1 µM pepstatin, and 0.125 units/µl benzonase) and incubated for 1 h at 4 °C. Subsequently, the cell lysate was centrifuged at 13,000 g, for 15 min at 4 °C, and the supernatants (protein extracts) were collected and stored at −80 °C. After extraction, the proteins were quantified using a Bradford assay and analyzed as previously described [8]. Briefly, 50 µg of each extract was concentrated, and the lysis buffer was exchanged with 50 mM ammonium bicarbonate using a 3-kDa ultra-filtration device (Millipore). The protein extract was denatured (0.1% *Rapi*GEST SF at 60 °C for 15 min) (Waters, Milford, USA), reduced using DTT (10 mM DTT at 60 °C for 30 min), alkylated with IAA (10 mM IAA at room temperature for 30 min in the dark) and enzymatically digested with trypsin (Promega, Madison, USA) at a 1:50 (w/w) enzyme:protein ratio. This reaction was terminated after the addition of 10 µL of 5% trifluoroacetic acid (TFA), and the internal standard, yeast alcohol dehydrogenase (P00330), was added to the digests to achieve 10 fmol μL^−1^ per injection [9], [10]. Qualitative and quantitative bi-dimensional nanoUPLC tandem nanoESI-HDMS^E^ experiments were conducted using a 1 h reverse-phase gradient from 7% to 40% (v/v) acetonitrile (0.1% v/v formic acid) at 500 nL min^−1^ on a nanoACQUITY UPLC 2D Technology system. A nanoACQUITY UPLC HSS T3 1.8 µm, 75 µm×15 cm column (pH 3) was used in conjunction with a reversed phase (RP) XBridge BEH130 C18 5 µm 300 µm x 50 mm nanoflow column (pH 10). A typical on-column sample load was 500 ng of total protein digests for each of the five fractions (500 ng/fraction/load). The resolving power for all measurements was at least 35,000 FWHM, and the ion mobility cell, filled with nitrogen gas, had a cross-section resolving power of at least 40 Ω/ΔΩ. The effective resolution with the conjoined ion mobility was >1,800,000 FWHM. The samples were ionized using a NanoLockSpray source (Waters, Manchester, UK) in the positive ion mode of nanoESI (+). The lock mass channel was sampled every 30 s. The mass spectrometer was calibrated with MS/MS spectrum of Glu-Fib peptide solution (100 fmol µL^−1^) delivered through the reference sprayer of the NanoLockSpray source. The doubly charged molecule [M + 2H]^2+^ = 785.8426 was used for initial single-point calibration and MS/MS fragment ions of Glu-Fib, such as a^+^, b^+^, and y^+^, were used to obtain the final instrument calibration. A Synapt G2-S HDMS instrument (Waters, Manchester, UK) was tuned to perform HDMS^E^ multiplexed DIA acquisition scanning with added specificity, orthogonality and selectivity and low and high collision energies in a T-wave transfer cell [11]. The mass spectrometer was automatically set to (a) switch between low energy MS (4 eV) and elevated collision energies HDMS^E^ (19–45 eV) applied to the transfer “T-wave” collision-induced dissociation (CID) cell filled with argon gas; and (b) adjust the trap collision cell to 1 eV for the total ion current. The scan time was previously adjusted based on the linear velocity of the chromatography peak delivered to the ion source to obtain a minimum of 20 scan points for each single peak, under both low-energy and high-energy transmission at an orthogonal acceleration time-of-flight (*oa*-TOF) and a range of 50–2000 m/z. The RF offset (MS profile) was adjusted, such that the nanoUPLC-HDMS^E^ data were effectively transmitted from 400 to 2000 m/z, ensuring that any masses observed in the high-energy spectra with less than 400 m/z arose from dissociations in the collision cell.

Protein identification and quantitative data packaging were performed as previously described [1], [12], [13], [14], [15]. A cut-off Log2ratio values of +1.0 (2-fold) or higher was applied for determining the proteins with higher abundance levels and −1.0 (2-fold) or lower for proteins with lower abundance levels to compare pairs of experimental groups [16], [17]. *In silico* prediction of protein-protein interaction and pathways of HUVEC proteins, with different abundance levels after interaction, was performed using QIAGEN׳s Ingenuity^®^ Pathway Analysis (IPA^®^, QIAGEN Redwood City, www.qiagen.com/ingenuity) and DAVID Bioinformatics Resources 6.7. The proteomic data were deposited in the ProteomeXchange Consortium [18] via the PRIDE [19] partner repository with the dataset identifier PRIDE: PXD002823.

The list of proteins with different abundance ratio filtered with 2-fold change criteria in *Δugm1 vs. c*ontrol, WT vs. control; *∆ugm1 vs.* WT; and GAG *vs.* control are presented in Tables 1–3. Pathways related to Δ*ugm1* (Δ*ugm1* vs. control and Δ*ugm1* vs. WT) interaction conditions are shown in Fig. 3. Furthermore, coincident pathways between Δ*ugm1* and GAG interaction conditions are shown in Fig. 4. Fig. 5 exemplifies one of these coincident pathways (*ILK signaling*).
